# Clonal T cell populations scarcely impair patients with rheumatic diseases: a prospective long-term follow up study

**DOI:** 10.1186/s13075-024-03444-0

**Published:** 2024-12-11

**Authors:** Michael Gernert, Tobias Müller, Lukas Schweiker, Marc Schmalzing, Matthias Fröhlich, Lea-Kristin Nagler, Patrick-Pascal Strunz, Hannah Labinsky, Eva Christina Schwaneck

**Affiliations:** 1 Department of Medicine II, Rheumatology and Clinical Immunology, University Hospital of Würzburg, Oberdürrbacher Str. 6, D-97080 Würzburg, Germany; 2 Chair of Bioinformatics, University of Würzburg, Am Hubland, D-97074 Würzburg, Germany; 3 Medizinisches Versorgungszentrum Rheumatologie und Autoimmunmedizin Hamburg GmbH, Mönckebergstraße 27, D-20095 Hamburg, Germany

**Keywords:** Rheumatoid arthritis, Spondyloarthritis, Monoclonal T cell populations, T-CUS, T-LGL, Long-term follow up, Infection

## Abstract

**Background:**

Clonal T cell populations are frequently detected in patients with rheumatic diseases. The relevance of this finding is often uncertain, as the clinical spectrum can range from being asymptomatic to T cell leukemia. Former studies suggested that certain anti-rheumatic drugs might influence the course of the clonal T cell populations.

**Methods:**

A prospective long-term follow up study was performed including patients with rheumatic diseases and clonal T cell populations. Clinical features, adverse events, especially infections and cytopenias, and immunosuppressive medication were assessed. T cell populations were characterized by polymerase chain reaction, flow cytometry and stimulated cell cultures.

**Results:**

28 Patients with rheumatoid arthritis, spondyloarthritis, or giant cell arteritis were prospectively followed for up to 7.6 years. Severe infections or cytopenias (10.7% autoimmune neutropenias) were rare. The clonal T cell populations mostly persisted over time, the tumor burden decreased in the long-term. The cytokine secretion in stimulated T cell cultures did not differ in the subgroup of RA patients with versus without clonal T cells.

**Conclusion:**

Clonal T cell populations in patients with rheumatic diseases are common, but are rarely harmful. Feared neutropenia, infections or progression into T cell leukemia could not be detected in the long-term in our cohort.

**Supplementary Information:**

The online version contains supplementary material available at 10.1186/s13075-024-03444-0.

## Background

Clonal T cell populations have been described in several autoimmune conditions such as rheumatoid arthritis, systemic lupus erythematosus or autoimmune cytopenias, but can also be present in healthy individuals [1]. Clonal T cell populations, which do not cause clinical problems can be named as T cell clones of uncertain significance (T-CUS) [2]. The clonal T cells are thought to arise from chronic antigenic stimulation and dysregulation of apoptosis due to activation of survival pathways. If the monoclonal T cells exceed 0.5 × 10^9^ cells per liter in the peripheral blood or become clinically apparent, they are called T cell large granular lymphocyte (T-LGL) leukemia [3]. T-LGL-leukemia is a chronic mature lymphoproliferative disorder involving large, granular T-cells lasting for more than six months. Natural killer (NK)-cell variants are usually not associated with autoimmune diseases and will not be discussed here. T-LGL-leukemia and T-CUS can be difficult to distinguish in clinical routine. LGL cells per se are part of the normal peripheral blood mononuclear cells (comprising 10–15%), are cytotoxic T cells or NK cells, they are polyclonal, and their presence in the peripheral blood is not pathological [4]. To prove clonality polymerase chain reaction (PCR) is performed and to characterize the T cell population flow cytometry is applied. Most commonly the clonal T-cells have the following phenotyope: CD3^+^/CD8^+^/CD57^+^/αβT-cell receptor (TCR)^+^, whereas less frequent variants include γδTCR^+^ and CD4^+^/CD8^−^ variants [5].

Data from a Dutch nationwide population-based cancer registry led to an estimated overall age-standardized incidence rate of T-LGL-leukemia of 0.72 per 1.000.000 person-years [6]. Up to 74% of T-LGL-leukemia patients suffer from autoimmune diseases, the most common of which is rheumatoid arthritis (RA) [7]. Felty’s syndrome, which consists of RA, splenomegaly, and neutropenia, is associated with clonal T-LGL populations in up to a third of patients. The two diseases might actually be different manifestations of the same entity. In the past, T-LGL leukemia may often have been overlooked in Felty’s syndrome cohorts, when TCR-PCR was not performed. Therefore, LGL-count and clonality assessment of T cells are necessary for differentiation [3, 8].

In 2015, we described the prevalence of clonal T-LGLs in a large RA cohort with 3.6%. Furthermore, we found an association between therapy with TNFα inhibitors (TNFi) and clonal T cell populations [9]. Data about further risk factors for T-LGL populations is scarce. TNFi therapy (in combination with azathioprine) is associated with hepatosplenic γδT-cell lymphomas in patients with Crohn’s disease [10]. TNFi might play a role in the expansion of clonal γδT-cells in patients with psoriasis, as γδT-cell counts increase after infliximab infusions [11]. But also under various immunosuppressive medications, due to solid organ transplantation, T-LGL leukemias are described [12].

Therefore, the aim of the present study was to perform a long-term follow up study for up to 7.6 years to characterize the clinical implications, the appearance of infections and neutropenias, and the impact of the immunosuppressive therapies in patients with autoimmune diseases and clonal T cell populations.

## Patients and methods

### Patients and definitions

Twenty-eight patients were recruited for this prospective long-term follow up cohort study at the rheumatologic outpatient department of the university clinic of Würzburg between 2014 and 2019. The patients were identified as having clonal T cells within the formerly described T cell clonality prevalence studies from Schwaneck et al. [9, 13] The individuals who had a clonal T cell population in their peripheral blood were defined as having T cell clones of uncertain significance (T-CUS). Underlying diseases were RA (RA_+ T−CUS_), SpA (comprises axial spondyloarthritis and psoriatic arthritis), and giant cell arteritis (GCA) and fulfilled the respective ACR/EULAR classification criteria [14, 15] or ASAS classification criteria [16] for their rheumatic disease. Ten additional RA patients without clonal T cells (RA_noT−CUS_) were recruited as comparison group for morphologic and functional T cell experiments.

### Clonality analysis of the T-cell receptor, flow cytometry, and calculation of clonal T cell burden

PCR was performed to assess clonal rearrangement of the TCR-gene. This established method has been described elsewhere [17]. The clonal T cells were assigned to suspicious T cell populations, which were characterized by flow cytometry and laid within the γδT cell population (CD3^+^/γδTCR^+^; for an example see supplemental Figure S1) or in the NKT (CD3^+^/CD56^+^) cell population. The phenotype described in the literature being frequent in T-LGL leukemia (CD3^+^/CD8^+^/CD57^+^/αβTCR^+^) was here not the dominant population. Suspicious findings within these 2 populations were: Low or absent expression of CD2, CD5, or CD7; co-expression of CD4 and CD8, or neither of both; expression of CD57. Two panels were conducted using a 10 color cytometer (Navios 3L10c; Beckman Coulter, Krefeld, Germany): Panel 1 included CD45-KrO, CD3-PC5.5, CD56-PE, pan-γδTCR-FITC, CD4-APC, CD8-APCA750, CD2-PC7, CD5-ECD, CD7-PB; panel 2 included CD45-KrO, CD14-APCA700, CD56-PE, CD3-PC5.5, pan-γδTCR-FITC, CD4-APC, CD8-APCA750, CD57-PB. A third panel (to characterize the γδTCR domains) was additionally conducted only for the comparison experiments between RA_+ T−CUS_ and RA_noT−CUS_: Panel 3 included CD45-KrO, CD3-APCA700, pan-γδTCR-PC5.5, Vγ9-FITC, Vδ2-PB, Vδ1-PC7. All antibodies were purchased from Beckman Coulter (Krefeld, Germany). The sample preparation and calculation of cell numbers/µl has been described before [18].

### γδT-cell isolation, cell culture, and cytokine measurement

Only RA_+ T−CUS_ patients with their clonal T cell population within the γδT cell population were analyzed with the following functional assays and compared to RA patients without a clonal T cell population (RA_noT−CUS_). Peripheral blood mononuclear cells (PBMCs) were isolated with Ficoll-Paque Plus separation (GE Healthcare, Munich, Germany) and stored in liquid nitrogen before further processing. Magnetic cell sorting (MACS) with γδTCR monoclonal antibody-coupled microbeads (Miltenyi Biotec, Bergisch Gladbach, Germany) was performed twice (i.e. two consecutive columns) to achieve a sufficient γδT cell purity. Of these selected cells 1–2 × 10^5^ cells per well were stimulated with interleukin (IL)-2 (100 U/ml final concentration; Proleukin, Novartis, Nürnberg) + isopentenyl pyrophosphate (IPP) (10µM final concentration, Sigma Aldrich, Darmstadt) for 24 h at 37 °C. In the supernatants cytokines were detected with cytometric bead arrays (CBA flex set; BD bioscience, San Jose, CA) and measured with a LSR II cytometer (BD bioscience, San Jose, CA). To calculate cytokine concentrations FCAP array software (BD bioscience, San Jose, CA) was used. For each sample triplets were prepared and means were calculated.

### Statistical analysis

For statistical analysis, R version 4.3.2 (R Core Team (2023). R: A Language and Environment for Statistical Computing. R Foundation for Statistical Computing, Vienna, Austria) or SPSS Statistics v 28.0 (IBM, Armonk, New York) was applied. A *p*-value of < 0.05 (*) was considered statistically weak significant, *p* < 0.01 (**) significant, *p* < 0.001 (***) strong significant. To compare mean values between two groups, the Wilcoxon rank sum test was used, which is not based on normal distribution assumptions. To test for differences between unpaired groups Mann-Whitney U tests were performed for continuous variables and Pearson’s chi-squared test for categorical variables.

For the modelling of our patient data, we rely on mixed effect models [19]. These models are a type of statistical model especially useful for analyzing data with multiple levels of variability, like repeated measures or hierarchical structures. They extend linear models by incorporating both *fixed effects* (parameters that apply to the entire population) and *random effects* (parameters that account for individual differences). This dual structure is ideal for analyzing data from repeated measurements, such as longitudinal patient data. In longitudinal data, mixed effects models help capture both individual patient trajectories. Since each patient’s data involves repeated measures (e.g., at different time points), mixed effects models can account for the correlation between repeated observations within the same patient, which simpler models ignore.

Furthermore, we deal with count data, which we model by negative binomial regression, which can be interpreted as a variant or extension of ordinary gaussian regression. The latter model assumes that the outcome is continuous and normally distributed (including positive and negative outcomes), while count data are inherently discrete, positive and typically follow a distribution with a large proportion of small values (often including zeros). In particular, negative binomial regression models are designed specifically for such positive count data.

A model test for mixed effect models was performed to analyze several parameters which should be integrated into the model. In detail, the *step* method was applied as implemented in the lmerTest R package [20] which is applicable for mixed effect models. This function contains on the one hand the *drop1* function for fixed effects that computes the F-test for all marginal terms that can be dropped from the model while respecting the hierarchy of terms in the model. For random effects we relied on the function *ranova*, which computes an ANOVA-like table with tests of random-effect terms in the model. Each random-effect term is reduced or removed, and likelihood ratio tests of model reductions are calculated as in the case of the function *drop1*. We started with a saturated model which was pruned in the following until no feature could be dropped from the model. This final model is reported. The fitted models were visualized by functions implemented in the R package sjPlot [21].

## Results

### Patients’ characteristics

#### Clinical characteristics of the study cohort

Twenty-eight persons with clonal T cell populations were included in the study with a median observation time of 56.5 months (range 18–91). The median age was 62.5 years (range 43–87) and 16 (57.1%) were female. Included were 19 RA patients, 7 SpA patients, 1 with GCA and 1 person without a rheumatic disease, with a disease duration before baseline of 14.0, 10.0, or 1.0 years, respectively. All patients took DMARDs before inclusion in the study. Regarding disease activity, the median DAS28-CRP during the study was 2.5 (IQR 2.0-2.9) in the RA patients and the median BASDAI was 4.1 (IQR 2.3–6.4) in the SpA patients. 16/19 (84.2%) RA patients ever took TNFi with a duration of use of 2.0 years (IQR 0.1–12.0), 6/7 (85.7%) SpA patients ever took TNFi with a duration of use of 9.4 years (IQR 6.5–12.0). The median daily prednisolone dose was 0.1 mg (IQR 0.0–2.0) in the RA patients, 0.0 mg (IQR 0.0-1.8) mg in the SpA patients, and 1.1 mg in the GCA patient (Table 1).

**Table 1:** Characteristics of the study population

	Whole population (*n* = 28)	RA patients (*n* = 19)	SpA patients (*n* = 7)	Other (1 GCA and 1 HD)
**Clinical characteristics**				
Observation time, median (range), months	56.5 (18–91)	74.0 (23–91)	32.0 (18–42)	32 and 39
Female, *n* (%)	16/28 (57.1)	14/19 (73.7)	1/7 (14.3)	1/2 (50.0)
Age, median (range), years	62.5 (43–87)	62.0 (43–87)	65.0 (50–82)	64.5 (55–74)
Disease duration, median (IQR), years	12.0 (7.0–24.0)	14.0 (7.0–24.0)	10.0 (9.0–28.0)	1.0*
Disease activity during study, median (IQR), points	na	DAS-28-CRP: 2.5 (2.0-2.9)	BASDAI: 4.1 (2.3–6.4)	na
Daily prednisolone dose during study, median (IQR), mg	0.03 (0.00-1.63)	0.07 (0.00-1.99)	0.00 (0.00-1.80)	1.13 and 0
TNFi intake ever, *n* (%)	22/28 (78.6)	16/19 (84.2)	6/7 (85.7)	na
Duration of TNFi intake, median (IQR), years	4.3 (0.1–12.0)	2.0 (0.1–12.0)	9.4 (6.5–12.0)	na
**T cell characteristics**				
Timespan between 1st and last TCR-PCR, median (IQR), years	3.4 (2.2–5.9)	5.6 (3.3–6.1)	2.0 (1.7–2.4)	2.2 and 3.9
Main flow cytometric phenotype, *n* (%) CD3^+^/γδT^+^ CD3^+^/CD56^+^ (NKT)	20/28 (71.4) 8/28 (28.6)	13/19 (68.4) 6/19 (31.6)	6/7 (85.7) 1/7 (14.3)	1/2 (50.0) 1/2 (50.0)
Coexpression of surface markers, *n* (%) CD57^+^ CD4^+^/CD8^−^ CD8^+^/CD4^−^ CD4^+^/CD8^+^ CD4^−^/CD8^−^	19/28 (67.9) 0/28 (0.0) 9/28 (32.1) 7/28 (25.0) 12/28 (42.9)	12/19 (63.2) 0/19 (0.0) 7/19 (36.8) 6/19 (31.6) 6/19 (31.6)	5/7 (71.4) 0/7 (0.0) 2/7 (28.6) 0/7 (0.0) 5/7 (71.4)	2/2 (100.0) 0/2 (0.0) 0/2 (0.0) 1/2 (50.0) 1/2 (50.0)
Clonal T cell burden during study, median (IQR), cells/µl	250.0 (149.5-381.3)	261.0 (143.0-410.0)	249.0 (105.0-358.0)	169.0 and 468.0

* includes only the GCA patient, not the HD. *GCA* giant cell arteritis, *HD* healthy donor, *IQR* interquartile range, *na* not applicable, *NKT* natural killer T cells, *PCR* polymerase chain reaction, *RA* rheumatoid arthritis, *SpA* spondyloarthritis, *TCR* T cell receptor, *TNFi* tumor necrosis factor alpha inhibitor

#### 3.1.2 Characterization of T cell properties

All participants’ peripheral blood was analyzed at least twice with PCR of the TCR. The median timespan between the first and last PCR was 40.7 months (IQR 25.8–71.8). In flow cytometry all patients exhibited CD3^+^, and either coexpressed γδTCR (71.4%) or CD56 (28.6%). A common coexpressed surface marker was CD57 (67.9%), CD4^−^/CD8^−^ in 42.9%, CD8^+^/CD4^−^ in 31.1%, and CD4^+^/CD8^+^ in 25.0%. The median clonal T cell burden during the study was 250.0/µl (IQR 149.5-381.3).

### 3.2 Declining, but persisting clonal T cells in the long-term

TCR-PCR was performed at least twice during the follow up and revealed persistence of the clonal T cell population in 27/28 (96.4%) persons. In 1 RA patient in the second TCR-PCR the clonality was no longer detectable after 38 months. 24 persons exhibited 1 clonal peak in the TCR-PCR (monoclonal), 4 persons showed 2 peaks (biclonal).

**Fig. 1:**
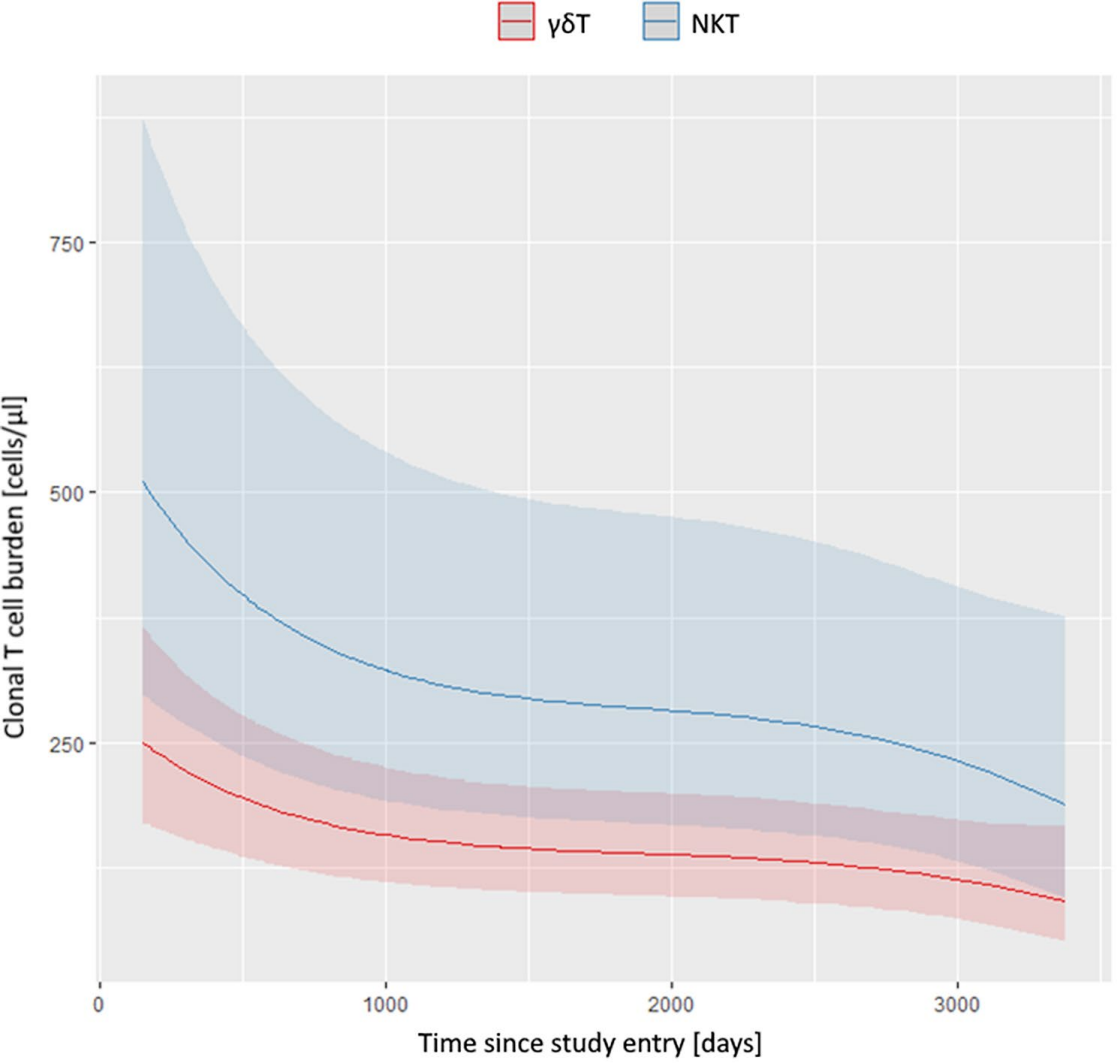
Course of clonal T cell burden over time predicted by a negative binominal mixed effect model. Patients having a clonal NKT cell population are shown in blue, having a clonal γδT population are shown in red (each with 95% confidence bands)

At baseline the median clonal T cell burden was 249.0 (IQR 182.8–444.0) cells/µl and at the last visit 158.5 (81.8-357.5) cells /µl. The optimal model for clonal T cell burden over the time course depends only on the parameters time and T cell group (NKT cells or γδ T cells). The statistical model confirmed that the clonal T cell burden decreased over time (Fig. 1). The model test starts with the full saturated model with the following parameters, which were implemented to test for their influence on the clonal T cell burden: TNFi treatment, infections, neutrophil count, T cell group (i.e. NKT or γδT), and prednisolone dose. None of these parameters influenced the clonal T cell burden in the time course, except the T cell group (*p* = 0.034), as the initial NKT cell counts were usually higher than γδT cell counts. This difference persisted over time (Fig. 1).

### 3.3 TNFi treatment does not affect the clinical course and burden of clonal T cells

Of the 28 patients, a total of 318 measurements were evaluated, 98 of which were under TNFi treatment. With a simple Mann-Whitney U test a significant difference of the clonal T cell burden between TNFi and no TNFi appeared to be present (supplemental Figure S2). To take the repeated measurements per patient into account a more elaborated statistical model was needed, why a regression model was applied. This showed that TNFi treatment did not influence the tumor burden over time (*p* = 0.208).

### 3.4 Little clinical impact of clonal T cell populations

The clinical relevance of the clonal T cells was investigated with the model testing for the mixed effect model to correlate the prevalence of infections in dependence of TNFi intake, tumor burden, neutrophil count, T cell group, and prednisolone dose. As the optimal model excluded all parameters, no association between the prevalence of infections and the aforementioned parameters could be detected.

Infectious complications during the observation period were scarce. Altogether 52 infections in 17 patients requiring antibiotic treatment occurred (0.4 per year observed). Among these infections, 5 were pneumonias in 5 patients accounting for 0.04 pneumonias per year and 1 septic arthritis (0.008 per year). No herpes zoster reactivations or thrush were documented.

Three patients (all RA) developed neutropenia during the study, and 1 autoimmune hemolytic anemia, 4 new onset splenomegalies, 1 thrombosis, and 1 malignancy (pancreatic cancer) occurred. One SpA patient died after a fall/trauma (Table 2).

**Table 2:** Adverse events during long-term observation

	Whole population (*n* = 28)	RA patients (*n* = 19)	SpA patients (*n* = 7)	Other (1 GCA and 1 HD)
**Infectious events**				
Infections requiring antibiotic treatment total events incidence per year per patient, median (IQR)	52 0.28 (0.00-0.74)	44 0.29 (0.00-0.72)	7 0.00 (0.00-0.92)	1 0.00 and 0.31
Pneumonia total events incidence per year per patient, median (IQR)	5 0.00 (0.00–0.00)	5 0.00 (0.00-0.13)	0	0
Septic arthritis total events incidence per year per patient, median (IQR)	1 0.00 (0.00–0.00)	1 0.00 (0.00–0.00)	0	0
**Autoimmune and other events**, ***n*(%)**				
Autoimmune neutropenia	3/28 (10.7)	3/19 (15.8)	0/7 (0.0)	0/2 (0.0)
Autoimmune hemolytic anemia	1/28 (3.6)	1/19 (5.3)	0/7 (0.0)	0/2 (0.0)
New onset of splenomegaly	4/28 (14.3)	3/19 (15.8)	1/7 (14.3)	0/2 (0.0)
Malignancy	1/28 (3.6)	1/19 (5.3)	0/7 (0.0)	0/2 (0.0)
Deep vein thrombosis	1/28 (3.6)	1/19 (5.3)	0/7 (0.0)	0/2 (0.0)
Death	1/28 (3.6)	0/19 (0.0)	1/7 (14.3)	0/2 (0.0)

GCA giant cell arteritis, HD healthy donor, RA rheumatoid arthritis, SpA spondyloarthritis

### Morphological comparison of lymphocyte subsets between RA patients with or without clonal T cells

To characterize the properties of the clonal T cell populations, a RA population was selected with clonal T cells (RA_+ T−CUS_) and was compared with a matched RA group without T cell clonality (RA_noT−CUS_). All patients appeared to have their clonal T cells within the γδT cell population. The patients’ characteristics and comparison between these groups are shown in supplemental table S1.

Lymphocyte subsets in the peripheral blood were similar distributed comparing RA_+ T−CUS_ to RA_noT−CUS_ patients, apart from lower T helper cells (CD3^+^/CD4^+^) in RA_+ T−CUS_ (784.5 [391.9–1083.0]/µl versus 1109.2 [813.7-1689.1]/µl, *p* = 0.031). Regarding γδT cell subsets (i.e. Vγ9-, Vδ2-, or Vδ1-domaine), also no differences were detected between RA_+ T−CUS_ and control patients (RA_noT−CUS_) (Table 3).

**Table 3:** Flow cytometric comparison of lymphocytes between RA patients with versus without clonal T cells

	RA + clonal T cells (RA_+ T−CUS_) (*n* = 14)		RA without clonal T cells (RA_noT−CUS_ = control) (*n* = 10)		*p*-value comparing cells/µl
	Percentage	Cells/µl	Percentage	Cells/µl	
Lymphocytes	23.1 (17.1–30.0)^§^	1790.0 (910.0-2140.0)	26.8 (22.5–33.5)^§^	1900.0 (1482.5–3370.0)	0.259
*Within the lymphocyte gate*					
T cells (CD3^+^)	72.8 (67.9–82.3)	1370.0 (702.9-1802.7)	75.8 (68.5–81.7)	1509.4 (1108.4-2596.1)	0.285
T helper cells (CD3^+^/CD4^+^)	45.2 (38.7–51.4)	784.5 (391.9–1083.0)	55.9 (46.6–61.8)	1109.2 (813.7-1689.2)	0.031*
Cytotoxic T cells (CD3^+^/CD8^+^)	24.3 (17.7–34.4)	412.8 (177.1-674.9)	18.8 (13.3–25.9)	318.1 (238.9-564.4)	0.886
CD4/CD8 ratio	1.74 (1.27–2.45)		2.91 (1.87–4.20)		0.138
NK cells (CD3^−^/CD56^+^)	13.7 (9.0-23.2)	217.9 (122.4-327.2)	10.6 (4.6–14.3)	227.3 (122.2-345.2)	0.122
NKT cells (CD3^+^/CD56^+^)	10.0 (4.0-14.1)	100.6 (49.0-348.6)	3.6 (1.4–8.8)	89.4 (28.1-135.4)	0.285
Pan-γδT cells	6.9 (3.0-6.5)	91.3 (72.5-356.5)	4.4 (3.0-6.5)	85.0 (58.8-107.8)	0.437
*Within the γδT cell gate*					
Vγ9	73.8 (12.4–94.2)	66.0 (10.5-263.3)	80.6 (58.1–92.9)	61.9 (33.3-103.4)	0.931
Vδ2	68.8 (19.0–93.0)	61.2 (12.9-251.2)	80.6 (58.1–92.9)	61.5 (32.0-102.6)	1.000
Vδ1	19.9 (3.1–71.1)	24.9 (2.9–67.0)	13.3 (5.0-30.6)	13.7 (4.7–18.6)	0.472

*significant < 0.05 in a Mann-Whitney U test. ^§^in the differential blood count. *T-CUS* T cell clones of uncertain significance

### Functional comparison of clonal T cells versus polyclonal T cells in RA patients

To characterize the functional aspects of the γδT cells cytokine secretions were measured in γδT enriched cultures (purity > 90%), which were stimulated with IL-2 and IPP for 24 h. Most cytokines were not detectable without stimulation (supplemental Table S2).

**Fig. 2:**
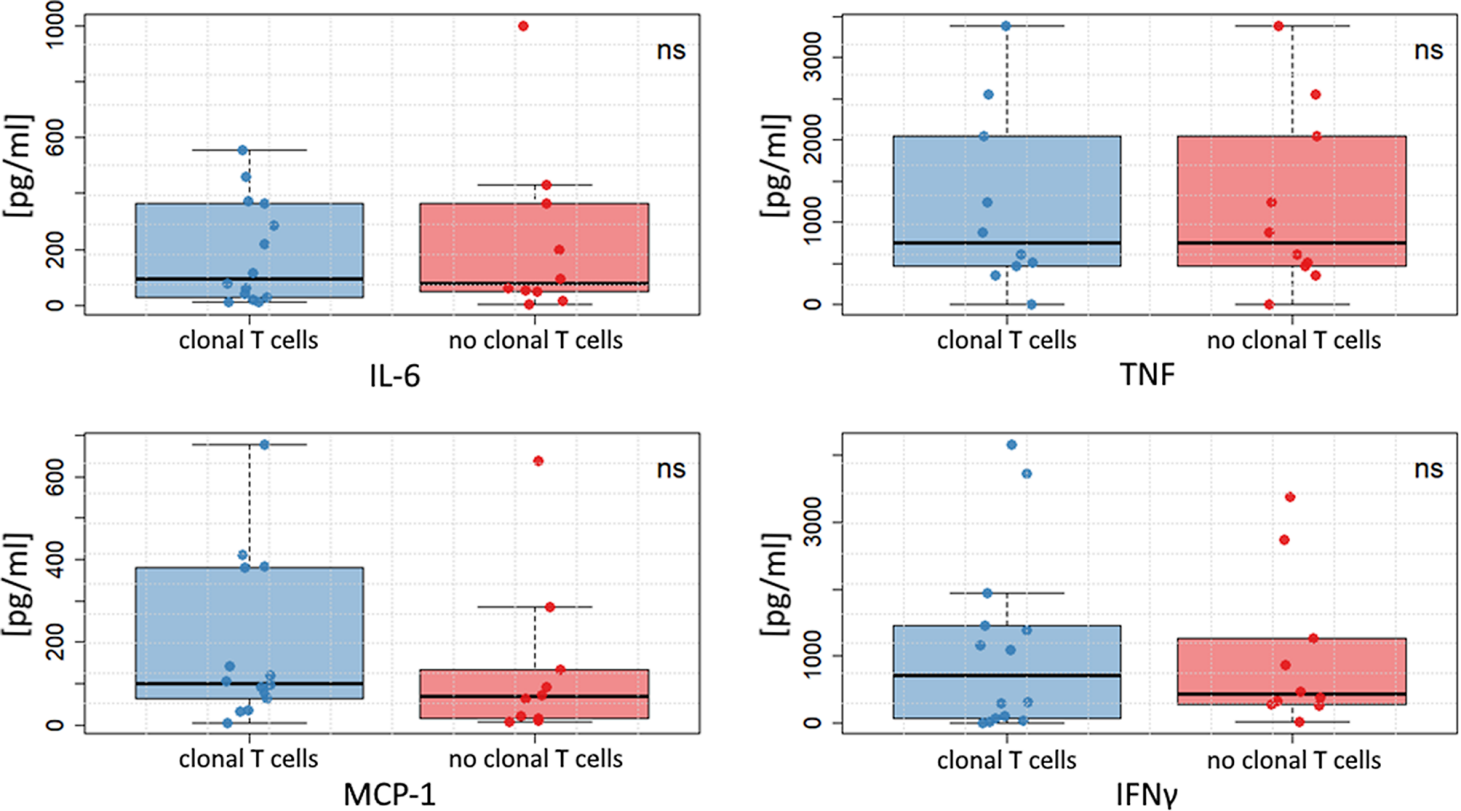
Comparison of cytokine secretions in stimulated γδT cell cultures from RA patients with clonal T cells (RA_+ T−CUS,_ blue boxes) versus without clonal T cells (RA_noT−CUS_, red boxes). In all comparisons no significant differences were identified. *IL* interleukin, *IFN* interferon, *MCP* monocyte chemoattractant protein, *ns* not significant, *TNF* tumor necrosis factor

Upon stimulation the following cytokines could be detected in the supernatants of the γδT cell cultures: IL-6, TNF, monocyte chemoattractant protein (MCP)-1, and interferon (IFN)γ. No differences were detected in these cytokines between RA_+ T−CUS_ versus RA_noT−CUS_ patients (IL-6: 77.6 pg/ml [IQR 39.4-379.9] vs. 96.2 [26.9-366.1], *p* = 0.931; TNF: 746.3 [444.2-2171.7] vs. 640.2 [82.9-1645.8], *p* = 0.666; MCP-1: 68.6 [14.9-172.8] vs. 100.5 [57.7-380.4], *p* = 0.235; IFNγ: 426.2 [279.1-1636.9] vs. 706.6 [66.1–1579.0], *p* = 0.931) (Fig. 2).

## Discussion

We describe a prospective cohort of 28 subjects, mostly with rheumatic diseases, having clonal T cell populations and followed them prospectively for up to 7.6 years. A relevant tendency to infections or neutropenia was not present. Clinically manifest T cell lymphoma or leukemia did not occur. Overall, the cohort was little impaired despite the presence of clonal T cells. Morphologic or functional differences between RA patients with clonal T cells versus RA without clonal T cells were not detected.

To our knowledge this is the first prospective long-term follow up of patients with clonal T cells and rheumatic diseases. Our motivation for this study was the fact, that detection of clonal T cells often leads to concerns about its significance and prognosis in patients and practitioners, although clonal T cells are described in various conditions. Next generation sequencing of the TCR showed that patients with RA have an increased number of clonally expanded T cells compared to patients at risk for RA [22]. Clonal T cells are already present at initial diagnosis in RA, but also in healthy persons [23]. Furthermore, in SLE, inflammatory bowel disease, and psoriasis clonal T cell populations are described [24–26].

It is thus of vital importance to discriminate between clonal T cells without current clinical affection, a condition labeled as T-CUS, and T-LGL leukemia or Felty’s syndrome, as the latter two often overlap and coincide with severe symptomatic neutropenia [27]. Felty’s syndrome per se is not defined by clonality of the TCR. At study entry our patients did not exhibit symptoms of T-LGL leukemia or Felty’s syndrome, hence they were classified as having T-CUS. Morphological and functional tests of the suspicious T cell population were not different between RA patients with clonal T cells versus without clonal T cells. Patients with clonal T cells proven by PCR and attribution to the γδT cell compartment, did not show differences or clonal expansions in the analyzed γ- or δ-domains of the TCR (i.e. Vγ9, Vδ2, or Vδ1). There were no differences in the γδT cell cytokine production. As T-CUS might be a pre-stage to T-LGL (and Felty’s syndrome), the long-term follow up of our T-CUS patients was important to us.

One of the most important outcome parameter in patients with T-LGL or Felty’ syndrome is the prevalence of infections. Infections in our cohort were scarce with 0.4 antibiotics per year observed, lying within the antibiotic prescription rates described for the general population (0.2–0.7 antibiotics per year) [28, 29]. We earlier reported a statistically significant association of anti TNF treatment and the clonal expansion of T-LGL cells. However, we did not find any connection between these therapies and the clonal T cell burden over time in this study [10, 14].

Treatment possibly influences the course of the clonal T cells. As most of our patients were treated intensively with DMARDs one might speculate that the sufficient treatment of the underlying rheumatic disease prevented further expansion of the clonal T cells. This assumption is supported by our finding that the clonal T cell burden decreased in the long-term. Similarly, the prevalence of Felty’s syndrome has declined since the introduction of efficacious DMARDs [30]. No treatment recommendations for T-CUS are available, and a specific treatment usually is not necessary, but different treatment regimens for T-LGL leukemia and Felty’s syndrome exist [31, 32]. In a population based study 45% of patients with T-LGL leukemia required systemic treatment [33]. Hence, for patients with clonal T cells treatment of the underlying rheumatic disease may be sufficient. During our clinical practice we have encountered several patients with RA und T-LGL-leukemia with severe neutropenia who profited from a therapy with rituximab. The T-cell clones of these patients did not differ in immune phenotype or tumor burden from the ones we describe in this prospective study.

The median tumor burden during our observation time was 250 cells/µl, thus not fulfilling the accepted definition of T-LGL leukemia requiring > 500 LGL cells/µl [34]. However, the high rate of immunosuppressive therapy might be responsible for a low clonal T cell burden in our cohort.

The clonal T cell burden can be followed up best by flow cytometry. In most of our patients an increased NKT cell or γδT cell proportion persisted and also in repeated PCRs a persistence of clonal T cells was present. As our patients did not exhibit significant clinical problems attributed to the clonal T cells in the long-term the question appears if regular follow-up investigations with PCR or flow cytometry are necessary, as they are often not part of clinical routine. Our data suggest, that monitoring frequency of infections (as the most important outcome parameter) and differential blood counts might be sufficient once a clonal T cell population is identified.

Limitations of our study are the single-center design, mainly comprising difficult to treat rheumatic diseases, and the small population size. Analysis of often mutated genes in T-LGL leukemia, like *STAT3*, would have been interesting, but was not done in our study. Our study cannot reveal the mechanisms behind clonal T cell persistence and mechanisms for progression of clonal T cells to T-LGL leukemia. A lager cohort should be analyzed in the future, including a prospective followed control group, to better compare infection rates and incidence of malignancies.

Summarized, at the beginning of our long-term follow up study a patient cohort with clonal T cells with uncertain significance was recruited. Our patients had persistence of their clonal T cells but did not develop significant cytopenias, infections or an increase of clonal T cells > 500/µl, which is the definition of T-LGL leukemia. The T cell clones in our cohort appeared to be *T cell clones without clinical significance* in the long-term. We speculate that clonal T cells scarcely become clinically apparent in patients with adequately treated rheumatic diseases even in the long-term and therefore our results might reassure practitioners and patients.

## Electronic supplementary material

Below is the link to the electronic supplementary material.

Supplementary figure S1: Example of a suspicious T cell population in flow cytometry. Elevated proportion of γδT cells within the lymphocyte gate (approximately 10% of lymphocytes) with co-expression of CD3^+^/γδTCR^+^/CD8^+^/CD57^+^ which was the most common pathological T cell population in our cohort. Not shown is exclusion of monocytes from the lymphocyte gate by CD14 staining.

Supplementary figure S2: Comparison of clonal T cell burden between patients treated with TNFi (red) versus no TNFi (blue). Within the no TNFi group many outliers were present, why the significant result in the shown Mann-Whitney U test needed further testing, as was done in the regression modelling.

## Data Availability

The data used and/or analyzed during the current study are available from the corresponding author on reasonable request.

## Abbreviations

ACR: American college of rheumatology
ASAS: Assessment of spondyloarthritis international society
CD: Cluster of differentiation
CRP: C-reactive protein
DAS: Disease activity score
DMARD: Disease modifying anti-rheumatic drug
EULAR: The European League Against Rheumatism
GCA: Giant cell arteritis
HD: Healthy donor
IL: Interleukin
IQR: Interquartile range
LEF: Leflunomide
MTX: Methotrexate
na: Not applicable
NK: Natural killer
PCR: Polymerase chain reaction
RA: Rheumatoid arthritis
SDAI: Simplified disease activity index
SpA: Spondyloarthritis
T-CUS: T cell clones of uncertain significane
T-LGL: T cell large granular lymphocytes
TCR: T cell receptor
TNFi: Tumor necrosis factor alpha-inhibitor
