# Supplementary figures and images for: Clonal T cell populations scarcely impair patients with rheumatic diseases: a prospective long-term follow up study
Source: Arthritis Res Ther. 2024 Dec 11;26:210. doi: 10.1186/s13075-024-03444-0 (PMC11633000; doi:10.1186/s13075-024-03444-0)

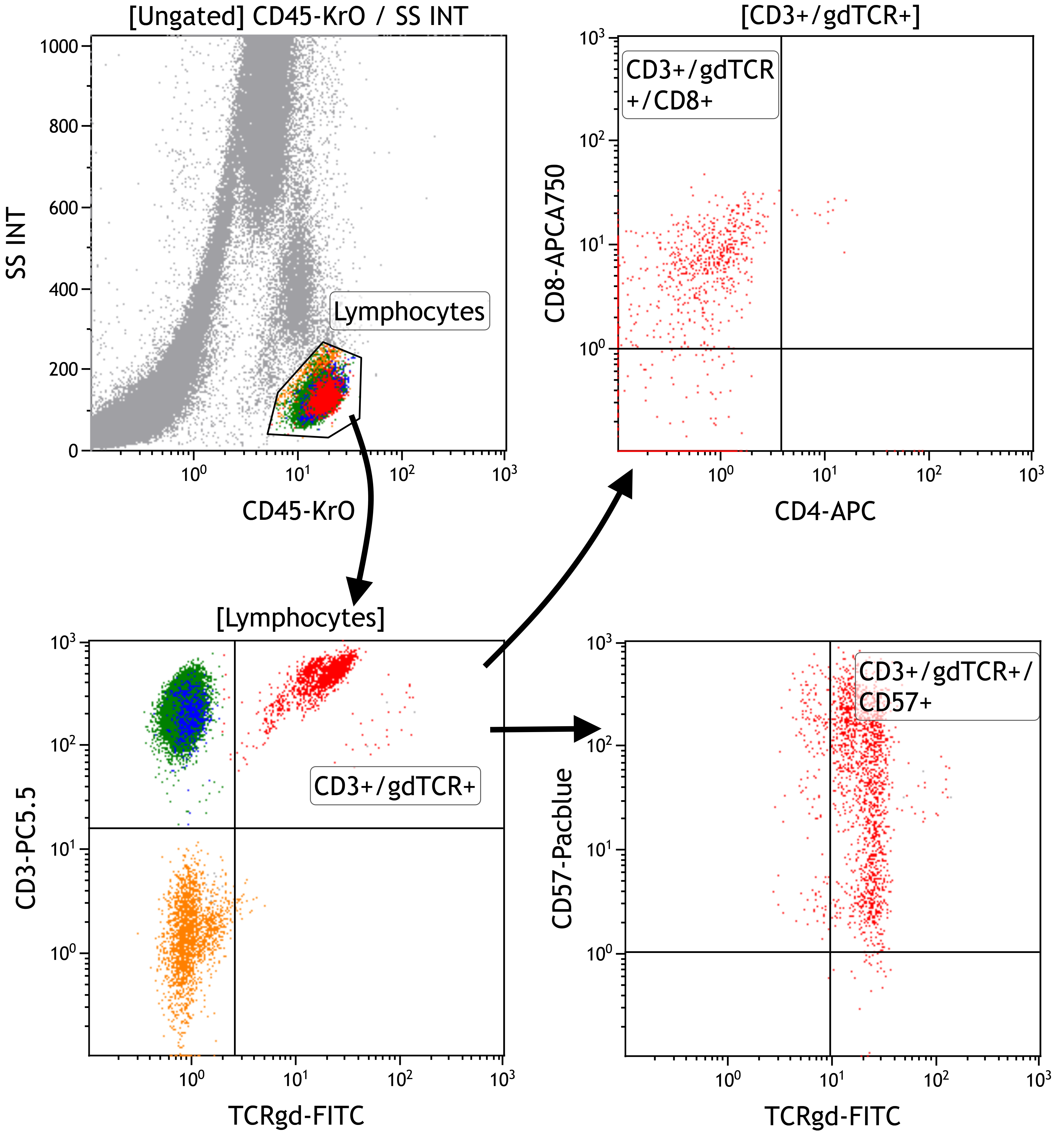

Supplement: Supplementary file 1 — Supplementary figure S1: Example of a suspicious T cell population in flow cytometry. Elevated proportion of γδT cells within the lymphocyte gate (approximately 10% of lymphocytes) with co-expression of CD3+/γδTCR+/CD8+/CD57+ which was the most common pathological T cell population in our cohort. Not shown is exclusion of monocytes from the lymphocyte gate by CD14 staining. [file 13075_2024_3444_MOESM1_ESM.tiff]

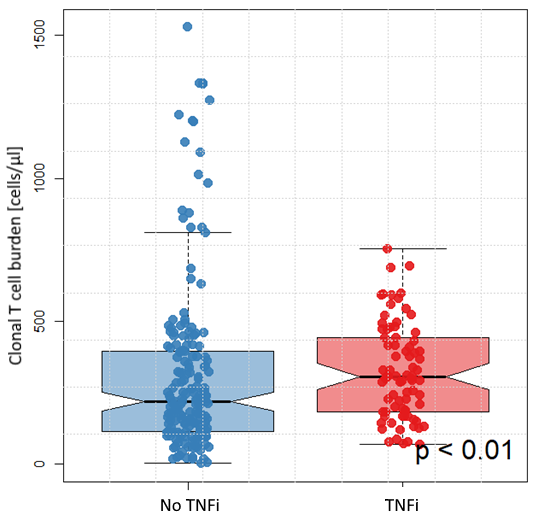

Supplement: Supplementary file 2 — Supplementary figure S2: Comparison of clonal T cell burden between patients treated with TNFi (red) versus no TNFi (blue). Within the no TNFi group many outliers were present, why the significant result in the shown Mann-Whitney U test needed further testing, as was done in the regression modelling. [file 13075_2024_3444_MOESM2_ESM.tiff]
